# Antibiofilm and Antivirulence Properties of 6-Polyaminosteroid Derivatives against Antibiotic-Resistant Bacteria

**DOI:** 10.3390/antibiotics13010008

**Published:** 2023-12-20

**Authors:** Delphine Vergoz, Hung Le, Benoit Bernay, Annick Schaumann, Magalie Barreau, Flore Nilly, Florie Desriac, Ali Tahrioui, Jean-Christophe Giard, Olivier Lesouhaitier, Sylvie Chevalier, Jean Michel Brunel, Cécile Muller, Emmanuelle Dé

**Affiliations:** 1 Univ Rouen Normandie, INSA Rouen Normandie, CNRS, Normandie Univ, PBS UMR 6270, F-76000 Rouen, France; delphine.vergoz@univ-rouen.fr (D.V.); hung.le1@univ-rouen.fr (H.L.); annick.schaumann@univ-rouen.fr (A.S.); 2 Univ Caen Normandie, Proteogen Platform, US EMERODE, F-14000 Caen, France; benoit.bernay@unicaen.fr; 3 Univ Rouen Normandie, Univ Caen Normandie, Normandie Univ, Communication Bactérienne et Stratégies Anti-Infectieuses, CBSA UR4312, F-76000 Rouen, France; magalie.barreau@univ-rouen.fr (M.B.); flore.nilly@umontpellier.fr (F.N.); florie.desriac@unicaen.fr (F.D.); ali.tahrioui@univ-rouen.fr (A.T.); olivier.lesouhaitier@univ-rouen.fr (O.L.); sylvie.chevalier@univ-rouen.fr (S.C.); 4 UNICAEN, Univ Rouen Normandie, INSERM, DYNAMICURE UMR 1311, F-14000 Caen, France; jean-christophe.giard@unicaen.fr; 5 Aix Marseille Univ, INSERM, SSA, MCT, F-13385 Marseille, France; bruneljm@yahoo.fr

**Keywords:** 6-aminosteroid derivatives, antivirulence, antibiofilm, pyocyanin

## Abstract

The emergence of multi-drug resistant pathogens is a major public health problem, leading us to rethink and innovate our bacterial control strategies. Here, we explore the antibiofilm and antivirulence activities of nineteen 6-polyaminosterol derivatives (squalamine-based), presenting a modulation of their polyamine side chain on four major pathogens, i.e., carbapenem-resistant *A. baumannii* (CRAB) and *P. aeruginosa* (CRPA), methicillin-resistant *S. aureus* (MRSA), and vancomycin-resistant *E. faecium* (VRE) strains. We screened the effect of these derivatives on biofilm formation and eradication. Derivatives ***4e*** (for CRAB, VRE, and MRSA) and ***4f*** (for all the strains) were the most potent ones and displayed activities as good as those of conventional antibiotics. We also identified 11 compounds able to decrease by more than 40% the production of pyocyanin, a major virulence factor of *P. aeruginosa*. We demonstrated that ***4f*** treatment acts against bacterial infections in *Galleria mellonella* and significantly prolonged larvae survival (from 50% to 80%) after 24 h of CRAB, VRE, and MRSA infections. As shown by proteomic studies, ***4f*** triggered distinct cellular responses depending on the bacterial species but essentially linked to cell envelope. Its interesting antibiofilm and antivirulence properties make it a promising a candidate for use in therapeutics.

## 1. Introduction

Gram-positive and Gram-negative pathogens exist as planktonic cells only at limited times during their life cycle. In response to environmental signals, pathogenic bacteria can adopt varied cellular fates, which involve the activation of virulence gene programs and/or the induction of a sessile lifestyle to form multicellular surface-attached communities called biofilms.

*Acinetobacter baumannii* and *Pseudomonas aeruginosa* are representatives of Gram-negative bacterial pathogens, both recognized as very problematic pathogens due to the spectacular increase of infections caused by multidrug-resistant (MDR) strains, in parti-cular, carbapenem-resistant strains [1]. The virulence of *A. baumannii* is a multifactorial process that does not rely on toxin production but rather on a “persist and resist” virulence strategy [2], where biofilm formation plays an important role. Several adhesins such as OmpA, Ata, Bap-like proteins, FhaC, tip pilin of fimbriae, the poly-N-acetylglucosamine polymer and capsular polysaccharides, and outer membrane (OM) vesicles, which all participate in biofilm formation, have been recognized as virulence factors [3]. Biofilms also participate in *A. baumannii* resistance against desiccation, disinfectants, or host immune systems. Reducing the formation of biofilms is one of the main ways of combating this pathogen. For its part, *P. aeruginosa* displays a large and complex arsenal of cell-associated (pili, secretion systems…) and extracellular virulence factors (toxins, enzymes…), which play an important role in colonization, bacterial survival, and tissue invasion. During acute infections, *P. aeruginosa* secretes virulence factors, including the phenazine pyocyanin that contributes to tissue damage and neutrophil defense inactivation [4,5]. Cell-associated factors are involved in biofilm formation, leading to chronic infections to avoid host defenses and compete with host microbiota [6].

*Staphylococcus aureus* and *Enterococcus faecium* are representatives of Gram-positive bacteria pathogens. *S. aureus* is a prominent human pathogen that causes systemic diseases, i.e., endocarditis, osteomyelitis, and bacteremia. These infections are often provoked by biofilms encountered on therapeutic devices. The pathogenicity of *S. aureus* mainly depends on the expression of multiple virulence factors, like hemolytic toxins, coagulase, and adhesion factors [7]. *S. aureus* shows an important ability to form biofilm on the surface of prosthetic devices. Thus, given the dissemination of methicillin-resistant phenotype (MRSA), the treatment of biofilm-associated infections is increasingly challenging. Unlike staphylococci, *E. faecium* does not produce pro-inflammatory toxins but synthetizes adhesion proteins that mediate adherence to host tissues [8] as well as biofilm formation [9]. Enterococci also have exceptional abilities to survive host or environmental stresses, as well as antimicrobial agents (like cephalosporin or aminoglycosides). They have the important ability to acquire and disseminate antibiotic-resistance determinants (like vancomycin-resistance genes for VRE strains), a malleable genome that can accumulate mutations, and exogenous genes that confer additional resistance [10]. *E. faecium* is intrinsically resistant to several antibiotics like ampicillin and aminoglycosides. Vancomycin is the usual alternative treatment for infections caused by ampicillin-resistant enterococci, but the emergence of high-level resistance has led to many therapeutic deadlocks [11].

For all these pathogens, the discovery of an effective antivirulence strategy could offer an interesting alternative to antibiotic treatments, whose effectiveness is diminishing.

**Figure 1:**
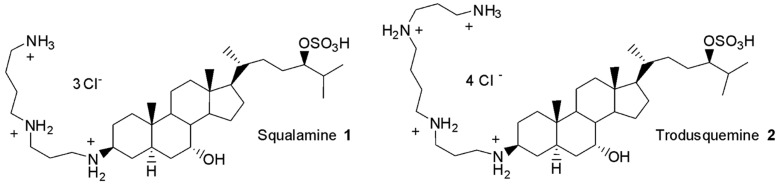
Structure of squalamine **1** and trodusquemine **2**.

In 1993, squalamine **1** and trodusquemine **2** (Figure 1) were identified as the first na-tural aminosterols from the dogfish shark, *Squalus acanthias*, to exhibit potent antimicrobial activities against both Gram-positive and Gram-negative MDR bacterial species [12,13].

Since then, polyaminosterol derivatives have been designed and evaluated for their antimicrobial activities, particularly due to their original mechanism of action [13,14]. Indeed, it was reported that in planktonic growth, the squalamine and claramine A1 derivatives have a bactericidal effect on Gram-positive bacteria via fast membrane depolarization and disruption [15]. On Gram-negative bacteria, a membrane permeabilization effect (similar to the one of antibacterial polymyxin [16]) associated with a disruption of the proton gradient makes these water-soluble compounds very promising therapeutics [15].

**Figure 2:**
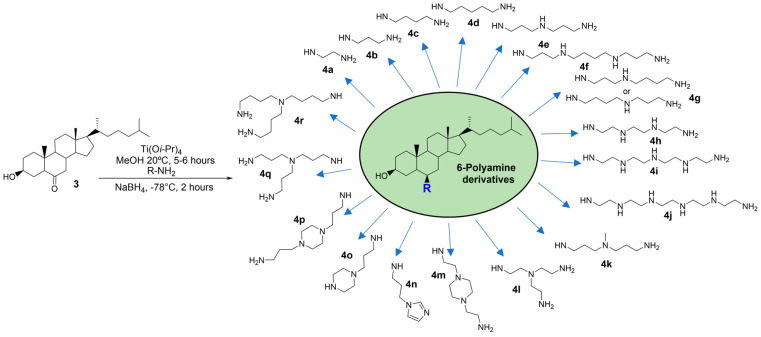
Scheme presenting the easily prepared 6-polyaminosteroid derivatives tested in this study. They are numbered from ***4a*** to ***4r*** according to the modification of their polyamine “R” side chain.

In a previous study, we easily prepared 6-polyaminosteroid compounds ***4a–4r*** (Figure 2) derived from squalamine that presented interesting antibacterial activities against both Gram-positive and Gram-negative MDR strains and were able to potentialize carbapenems, vancomycin, or oxacillin [17].

Here, to explore new routes to combat resistant bacteria, we examined the antibiofilm and antivirulence properties of these derivatives. Since biofilm is a common trait of the four pathogens tested, we first assayed their effect on biofilm formation and eradication for each strain. Then, for *P. aeruginosa*, which is known to produce numerous secreted virulence factors involved in acute infections, the derivatives were also tested for their effect on pyocyanin production. The capacity to lower the bacterial virulence *in vivo* using a *Galleria mellonella* infection model was observed in the most efficient antibacterial 6-polyaminosteroid ***4f*** derivative. Finally, to understand the mode of action of this derivative, we carried out proteomic experiments upon exposure to sub-inhibitory conditions and showed that the ***4f*** compound interfered with different cellular pathways according to the bacterial species.

## 2. Results and Discussion

To assay the antibiofilm and antivirulence properties of the promising antibacterial 6-polyaminosteroid derivatives [17], we used resistant archetype strains, i.e., a carbapenem-resistant *A. baumannii* (CRAB), a carbapenem-resistant *P. aeruginosa* (CRPA), a vancomycin-resistant *E. faecium* (VRE), and a methicillin-resistant *S. aureus* (MRSA).

### 2.1. Antibiofilm Activity of 6-Polyaminosteroid Derivatives

As described above, one of the virulence features common to all the pathogens is their ability to form biofilms. In this context, we evaluated the impact of 6-polyaminosterols on biofilms (Table 1) by measuring the biofilm prevention concentration (BPC) and the minimal biofilm eradication concentration (MBEC) of these compounds on the four resistant pathogens.

As shown in Table 1, 6-polyaminosteroid derivatives showed noticeable antibiofilm activities. Indeed, of the 19 derivatives tested, 5, 2, 14, and 16 prevented the formation of biofilms in CRAB, CRPA, VRE, or MRSA, respectively, with a BPC < IC_50_. BPC values were very close to the MIC values for all the molecules tested. This may be explained by the bactericidal activity of these compounds, previously demonstrated in [17], which allowed for the prevention of the 24 h biofilm formation as efficiently as the planktonic growth.

Biofilm eradication activity was then determined for derivatives presenting MIC < IC_50_. Interestingly, some of them allowed for the biofilm eradication of both Gram-negative and Gram-positive resistant bacteria (Table 1). For Gram-negative bacteria, the MBEC values of conventional antibiotics are usually higher than 256 mg/L [18,19,20]. The most active antibiotics on pre-formed biofilms are colistin and aminoglycosides, with MBEC values that can be as low as 32 mg/L on susceptible *A. baumannii* strains and 160 mg/L on *P. aeruginosa* [17,19,20]. *S. aureus* is also highly resistant in biofilm form, with an MBEC higher than 256 mg/L for antibiotics like oxacillin or around 32 mg/L for vancomycin [21]. Some *E. faecium* isolates can have MBEC values that can reach 128 mg/L for daptomycin, linezolid, or vancomycin [22].

Here, three of the tested compounds presented interesting activities on biofilms of CRAB: ***4e***, ***4r***, and ***4f*** with MBEC values of 32, 128, and 256 mg/L, respectively. ***4e*** had biofilm eradication activity similar to that of squalamine and, indeed, they were both as active as the best conventional antibiotics to eradicate biofilms. ***4r*** and ***4f*** possessed similar structures, with either an OH or MeO group in position 3 as well as spermine as a polyamine lateral chain and, interestingly, they had similar biofilm eradication activities. However, against *P. aeruginosa*, ***4f*** was the only derivative able to eradicate the biofilm at 256 mg/L. For *E. faecium*, ***4e*** and ***4f*** were also the most potent derivatives, with a biofilm eradication value of 64 mg/L, i.e., more effective than conventional antibiotics. Finally, against MRSA, four derivatives presented MBEC values below or equal to 128 mg/L, i.e., ***4b***, ***4d***, ***4e***, and ***4f***, and were even more active than squalamine for biofilm eradication. Altogether, ***4e*** (for CRAB, VRE, and MRSA) and ***4f*** (for all the strains) were the more potent 6-polyaminosterols to prevent or eradicate biofilms of these resistant species. It has to be noted that these compounds presented a norspermidine (***4e***) and a spermine (***4f***) lateral chain.

Since these 6-polyaminosteroid analogs of squalamine differed essentially by the flexible lateral chain, one can assume that the variations in MICs, BPC, or MBEC can be attributed at least partly to these moieties. Polyamines are small polycationic molecules involved in various bacterial physiological processes [23,24,25]. They can be involved in bacterial growth [26], surface motility, or siderophore synthesis; act as extracellular or intracellular signaling molecules; and regulate biofilm formation [24,27,28]. Numerous studies have reported that the addition of exogenous polyamines to biofilms results in various effects (inhibition or stimulation), depending on the bacterial species [24]. Here, a hypothesis of an effect of the polyamine side chain of derivatives on biofilms can be envisioned. Indeed, it was previously shown that *S. aureus* does not produce polyamines and presents sensibility to exogenous polyamines (inhibition of planktonic growth [27] at concentrations known to exist within human hosts [29]). A spermidine structural analog was also reported to inhibit biofilm formation and maintenance in *S. aureus* [30]. Polyamines production, like spermidine or norspermidine, is a species-level trait in enterococci and is encountered in *E. faecalis* but is rare in *E. faecium* (absent in our studied strain) [31]. This may explain the VRE hyper-susceptibility to the polyamines studied, like in *S. aureus* [29]. In *P. aeruginosa*, which is able to produce polyamines as putrescine or spermidine [32], the effect of exogenous polyamine addition is more mixed and depends on the polyamine tested. The putrescine was shown to promote biofilm formation by acting possibly as a host-associated signal that triggers bacteria lifestyle switching [33]; norspermidine was shown to inhibit biofilm formation and eradicate 24 h mature biofilm in *P. aeruginosa* PAO1 and clinical strains [34]. A similar effect of the polyamine lateral chain on *A. baumannii* biofilms is less likely. For this species, which only produces 1,3 diaminopropane but no polyamine with longer carbon chain, it was previously shown that the addition of diverse exogenous polyamines like spermine, spermidine, cadaverine, or putrescine did not impact biofilm development [35]. However, the amphiphilicity of the molecule associated with the positive charge of amines in precise spacing may act as a biofilm disruptor, as it was previously shown [30].

### 2.2. The Effect of 6-Polyaminosterols on CRPA Pyocyanin Production

To get further insights into the effects of 6-polyaminosterols on CRPA virulence, we assayed the production of one of its major secreted and soluble virulence factors, i.e., pyocyanin. This blue-green pigment is a redox-active phenazine that stimulates redox cycling in bacteria and enhances oxidative metabolism, which, in turn, increases the formation of intracellular reactive oxygen species (ROS) [36]. In the host, pyocyanin inhibits cell growth and ciliary movements and induces cell death and cytokine release [36]. The effect of 6-polyaminosterols on CRPA has been evaluated in a range of concentrations from 0.5 to 32 mg/L. As in the case of MBEC evaluation, we chose to focus on compounds, the MIC of which was below the IC_50_. Data represent the compound concentration leading to >40% and >70% reductions in terms of pyocyanin production (Table 2).

As shown in Table 2, each tested compound led to reduced pyocyanin production by at least 40%. The most efficient 6-polyaminosterol derivatives were indicated as reducing the pyocyanin by more than 70% at a concentration of 32 mg/L, except for compound ***4j***, which was capable of reducing pyocyanin by 70% at 16 mg/L. Accordingly, the polyaminoisoprenyl compound NV716 was shown to reduce pyocyanin production [37]. Since NV716 was also shown to reduce biofilm formation, it was suggested that reducing pyocyanin production decreased the release of a major matrix component of *P. aeruginosa* that is extracellular DNA [37]. Noticeably, the compounds that were the most efficient in pyocyanin production also displayed antibiofilm activity, suggesting that a similar mechanism could occur.

### 2.3. G. mellonella Treatment Assays

**Figure 3:**
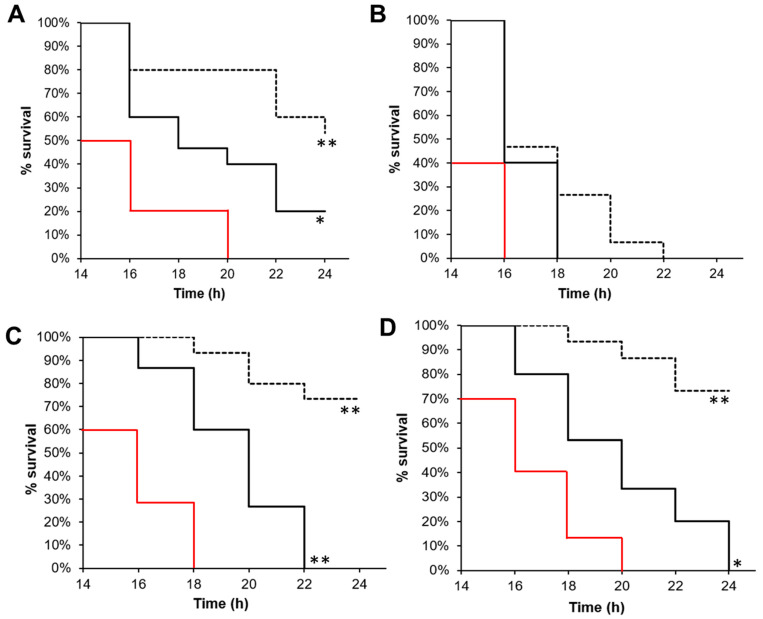
***4f*** and squalamine antivirulence activities. Survival curves of *G. mellonella* inoculated with (**A**) CRAB, (**B**) CRPA, (**C**) MRSA, or (**D**) VRE and treated 2 h post-infection with physiological solution (red line, control), ¼ MIC of squalamine (solid black line), or ***4f*** compound (dotted black line), as described in the methods section. Log-rank test * *p* < 0.01, ** *p* < 0.001.

The ***4f*** compound was the most potent derivative against all the studied strains. Consequently, we studied the effect of treatment with this compound or with squalamine against bacterial infections of *G. mellonella* larvae. *G. mellonella* provides an alternative model to study Gram-positive and Gram-negative bacterial infections. Like all insects, it lacks an adaptive immune response but possesses an innate immune response that shows remarkable similarities with that of vertebrates, allowing for an initial evaluation of antivirulence properties. The use of the *G. mellonella* model can consequently give indications about the appropriateness of compounds for therapeutic use. Larvae were infected with bacteria and, after 2 h, the ***4f*** or squalamine compounds were introduced separately at 14 mg/kg (or the equivalent of ¼ MIC). Larvae survival was then monitored for 24 h. As shown in Figure 3, the squalamine and ***4f*** compounds significantly prolonged the survival of larvae infected by CRAB, VRE, and MRSA during the first 20 h compared to the control. Moreover, the ***4f*** compound was more efficient than squalamine, with a survival rate ranging from 50% to 80% after 24 h against 0 to 20%, respectively. The equivalent of ¼ MIC of ***4f*** or squalamine was injected into the larvae, so the treatments were not able to inhibit bacterial growth. Consequently, the enhanced survival of larvae was not due to antibiotic activity but rather likely due to the antivirulence properties of this molecule. This is in accordance with data that previously reported that in *S. aureus* and *E. faecalis*, the reduced concentration of polyamines due to the activity of acetyltransferase increased bacterial survival within the host [38,39]. This conclusion is also supported by the fact that polyamines attenuate virulence in Gram-positive pathogens like *Streptococcus pneumoniae* [40] and could be used as a treatment for infectious diseases [25]. It must be noted that no significant effect was observed for CRPA.

### 2.4. Contributions to the Mechanism of Action of the **4f** Derivative

**Figure 4:**
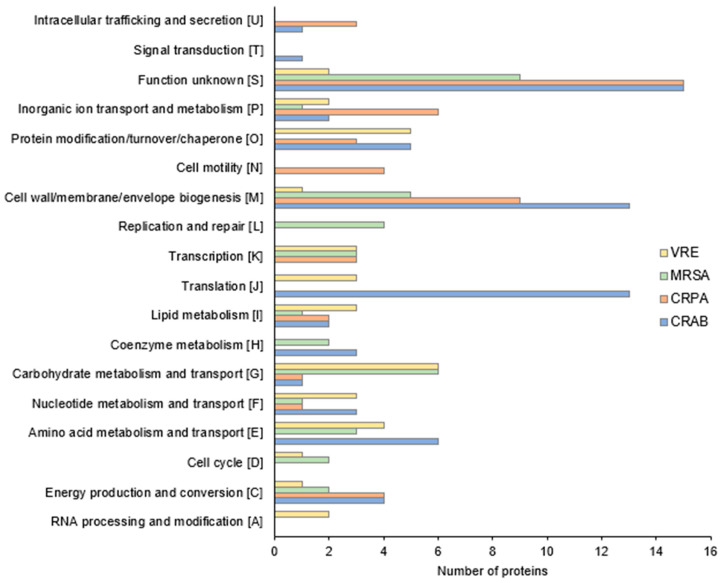
Functional COG classification of CRAB, CRPA, MRSA, and VRE differentially expressed proteins (fold change > 1.4, *p*-value < 0.05).

To gain a better understanding of how this particularly effective compound ***4f*** works, a label-free semi-quantitative proteomic analysis was used to compare the protein expression of the different bacterial species in the presence and absence of exposure to this molecule at a concentration equivalent to ¼ of the MIC. Differentially expressed proteins were characterized by a fold change value greater than 1.4, a *p*-value < 0.05, and then were categorized according to the Clusters of Orthologous Groups (COG, Figure 4). Following 2 h exposure to a ¼ MIC concentration, we identified 69, 35, 40, and 34 differentially expressed proteins in CRAB, CRPA, MRSA, and VRE, respectively (Figure S1).

Concerning Gram-negative bacteria, as shown in Figure 4 and Supplementary Tables S1 and S2, ***4f*** exposure induced notable alterations, primarily within the proteins responsible for the biogenesis of the cell wall and membrane, translation, and amino acid metabolic processes.

Interestingly, for *A baumannii*, we observed that the abundance of proteins involved in lipooligosaccharide biosynthesis (ABYAL2345, +2.6-fold; ABYAL3113, +1.6-fold) and OM lipoprotein transport (ABYAL3248, +1.5-fold) significantly increased following treatment with ***4f*** (Supplementary Table S1). These findings suggest that the principal mode of action of ***4f*** likely involves the OM destabilization of this Gram-negative bacterium through a mechanism resembling detergents [41]. A similar observation was noticed in *A. baumannii* when exposed to the cationic surfactants chlorhexidine and cetyltrimethylammonium bromide [42]. In line with this mode of action, the exposure of CRAB to ***4f*** also increased the abundance of ABYAL2090 (+4-fold) and ABYAL2091 (+4.5-fold) belonging to the multidrug efflux pump AdeABC (Table S1). A high level of transcription of this pump was indeed induced by benzalkonium and chlorhexidine [42]. These observations were therefore consistent with a mode of action via a detergent-like membrane permeabilization. In addition, proteins associated with oxidative stress defense (superoxide dismutase, +1.6-fold; NADH-quinone oxidoreductase, +1.5-fold; glutathione peroxidase, +1.5-fold; universal stress protein, +1.4-fold) and DNA repair (Excinuclease, +3.4-fold) were also increased in abundance. These proteins could be considered as the detoxification pathway of CRAB, limiting the entry of noxious compounds and the response to the oxidative damage of the cellular death pathway [43,44]. It is possible that ***4f*** exerted its lethal effect by additionally promoting the formation of harmful hydroxyl radicals, a common mechanism of cellular death underlying all classes of bactericidal antibiotics [45]. A decrease in the abundance of a cluster of ribosomal subunit proteins 30S and 50S (ABYAL3632, ABYAL3642, ABYAL2700, ABYAL3638, ABYAL3621, ABYAL0637) was also observed after ***4f*** treatment (Table S1 and Figure 4). These findings hint that ***4f*** did not only impact the cell wall but also potentially hindered the translation machinery of *A. baumannii*. This translation inhibition and the ability to perturb lipooligosaccharides could elucidate the reduction of *A. baumannii* virulence in the presence of ***4f***, as evidenced by the results observed in the *G. mellonella* experiments.

Proteomic investigations of the effects of ***4f*** on CRPA revealed 35 proteins whose abundance was affected (Supplementary Table S2). The most striking data for *P. aeruginosa* were that several proteins of the OM or that are involved in OM composition and/or modification were affected in response to ***4f*** exposure. We indeed noticed a lower abundance of six porins, among which were the substrate-specific proteins OprB, OpdC, OpdH, OprQ, and OprG; PhoP/Q; and low Mg^2+^ inducible OprH. Since *P. aeruginosa* displays 26 porins, our data indicate that about ¼ of these OM β-barrel proteins were less abundant in the CRPA envelope in response to ***4f***. Such a deep reduction in OMP production and/or abundance has been previously reported in *P. aeruginosa* that was subjected to cell envelope stress, as in the case of a *sigX* mutant [46,47]. SigX is an extracytoplasmic function sigma factor that is activated in response to membrane stresses [48,49]. In addition, ***4f*** exposure led to abundance alterations of proteins involved in the lipopolysaccharide (LPS) biosynthetic and/or modification pathways. Indeed, on one side, the LPS assembly protein LptD, lipid A biosynthetic acyltransferase HtrB1, and A-band LPS synthetic enzyme WbpW were reduced in response to ***4f*** treatment. Interestingly, a lower abundance of WbpW was shown to reduce the uptake of tobramycin [50]. On the other hand, L-Ara4N transferase ArnT was increased upon ***4f*** treatment. Noticeably, the *arn* cluster is involved in the aminoarabinose modification of LPS, thus decreasing the negative charge of the OM and limiting the uptake of cationic antimicrobials [51]. Altogether, these data suggest that ***4f*** exposure led to reduced LPS biogenesis and the modification of the LPS structure at the level of lipid A, leading possibly to adaptive resistance in a way that could be similar to that observed for polymyxins and cationic peptides [52]. It is thus conceivable that ***4f*** derivatives could disturb the OM by interacting with divalent cation binding sites on LPS, causing the disruption of these sites and promoting ***4f*** uptake, as was suggested for squalamine or claramine A1 [13,15]. This mode of action, different from the detergent-like permeabilization of *A. baumannii*, may originate from a different composition of the outer membranes of these bacteria as the OM of *P. aeruginosa* has LPS in its outer leaflet when lipooligosaccharide constitutes the one of *A. baumannii*.

For VRE, proteomic profiling showed that deregulated proteins were mainly involved in post-translational modification. More precisely, proteins implicated in proteolysis (UPI0001B6F78E, UPI00003C508F, UPI000037F70A) were considerably induced (Figure 4, Supplementary Table S3). We can also observe that important expression changes occurred for the metabolism proteins of carbohydrates (six proteins) and amino-acids (four proteins). We can thus hypothesize that the degradation products triggered by these catabolic activities could act in favor of the toxic effect of compound ***4f***. Interestingly, in VRE incubated in the presence of ***4f***, proteins involved in the osmotic stress response appeared to be less abundant. The amounts of the Na^+^/H^+^ antiporter NhaC and sodium/dicarboxylate symporter family protein, as well as two enzymes linked to the accumulation of osmoprotectant glycine betaine, were significantly reduced [53]. These data indicate that ***4f*** could impact the ability of *Enterococcus* to cope with osmotic challenge, which may contribute to its effects on virulence and/or antibacterial activity.

MRSA was the only strain showing the overproduction of proteins involved in DNA replication and repair (Figure 4). In addition, *ftsK* and *hup* gene products involved in chromosomal segregation and condensation, respectively, were also more abundant (Supplementary Table S4). Polyamines are positively charged molecules known to interact with DNA, but the correlation between the increased quantity of these polypeptides and the phenotypes observed when *Staphylococcus* is confronted with the ***4f*** molecule remains to be elucidated. However, the most spectacular overproduction observed when the MRSA cells were challenged with the ***4f*** compound was that of autolysin Atl. Peptidoglycan hydrolases (autolysins) are important enzymes in cell wall growth and turnover and cell separation, but they are also involved in antibiotic-induced lysis [54]. We can thus suspect that the deleterious effect of the ***4f*** molecule in *S. aureus* was linked to the induction of this lytic enzyme, as observed for penicillin. Of note, Szweda and collaborators proposed that autolysin can be used as a weapon against Staphylococci in combined therapy due to the enhanced antimicrobial activity of some antibiotics [55]. In this context, the ***4f*** molecule, in addition to its interesting antivirulent, antibacterial, and antibiofilm activities associated with the induction of autolysin production, could be a candidate as an “adjuvant” for treatment against MRSA.

In summary, our proteomic data revealed that the ***4f*** molecule triggered distinct cellular responses depending on the bacterial species. The higher number of differentially expressed proteins in Gram-negative bacteria, particularly *A. baumannii*, highlighted the flexibility of these pathogens to rapidly adapt to changing environments. Proteomic changes in the presence of ***4f*** within the Gram-negative group suggested a significant stress response in these bacteria, particularly affecting their cell envelope. Additionally, the observed alterations in translation, carbohydrate metabolism, and the autolysin process do not exclude them as additive action mechanisms of this compound on both Gram-negative and Gram-positive bacteria. These varied cellular response mechanisms may contribute to explaining the potential antivirulence effects observed in *G. mellonella* experiments. However, although our proteomic analysis has enabled us to gain a better understanding of the impact of compound ***4f*** on the overall physiology of the bacterial cell, the targets envisaged require confirmation by additional experiments at the molecular level.

## 3. Materials and Methods

### 3.1. The Synthesis of 6-Polaminosteroid Derivatives

All the compounds were prepared and fully characterized according to the procedure previously reported [17].

### 3.2. Bacterial Strains and Culture Conditions

The bacterial strains used in this study are listed in Table 3. Cultures of *S. aureus* and *E. faecium* were performed in brain–heart infusion (BHI) broth (Biokar diagnostics, Allonne, France) for preculture, and Mueller–Hinton MHB cation-adjusted (MHBII) (Biokar diagnostics) was used for biofilm formation and incubated at 37 °C without shaking. For *A. baumannii* and *P. aeruginosa*, biofilms were grown in MHBII in the dark at 37 °C without shaking.

### 3.3. Biofilm Prevention and Eradication Assays

For the determination of the BPC, biofilm formation was assessed in MHBII by the microtiter plate test in 96-well polystyrene plates, as previously described, with the following modifications [59]. For *S. aureus* and *E. faecium*, the inocula were adjusted to an OD600 nm of 0.2 in MHBII. The 6-polyaminosteroid derivatives (diluted in sterile ultra-pure water) were added at the same time as the inoculum. After 24 h at 37 °C for CRAB, CRPA, and MRSA and 48 h for VRE, biofilms were quantified by crystal violet assay [60]. BPC was defined as the first concentration of compound that prevented the detection of crystal violet staining (OD580 nm ≤ 0.1).

The MBEC for MRSA and VRE was assessed as previously described [60], with the following changes. Biofilms were exposed to 6-polyaminosteroid derivatives in MHBII for 24 h at 37 °C and then washed twice in saline solution (NaCl 0.9%) before the sonication of cells in fresh sterile MHB. The recovery plate was incubated at 37 °C for 24 h. OD600 nm was measured for each well to determine the MBEC value, defined as the lowest concentration of a compound that prevented measurable growth in the recovery medium [61]. For CRAB and CRPA, the MBEC was determined as previously described [20].

All assays were performed at least in duplicate in a minimum of three independent experiments.

### 3.4. Pyocyanin Quantification Assays

CRPA was grown in MHBII from an initial inoculum adjusted to an OD580 nm of 0.08 in a 96-well microtiter plate at 37 °C for 24 h with shaking (180 rpm). After incubation for 24 h, cell growth was determined by measuring OD580 nm. The pyocyanin quantification assay was carried out as described previously [62,63]. Briefly, one volume of chloroform was used to extract free-cell supernatant samples. Then, ½ volume of 0.5 M HCl was added to the chloroform layer (blue layer). The absorbance of the HCl layer (red-pink layer) was recorded at 520 nm using the Spark 20 M multimode microplate reader controlled by SparkControlTM software Version 2.1 (Tecan Group Ltd., Männedorf, Switzerland) and the data were normalized for bacterial cell density (OD580 nm).

### 3.5. Antivirulence Assays

The protocol used for these assays was inspired by [64]. Briefly, bacterial strains were cultured in BHI, harvested at OD600 = 0.6, and washed twice in 0.9% NaCl. Cultures were adjusted to reach a density of 2.10^8^, 2.10^9^, 1.10^8^, and 5.10^6^ CFU/mL for MRSA, VRE, CRAB, and CRPA, respectively. Fifteen randomly selected *G. mellonella* larvae around 280 mg were used for each test group. A 10 µL, an inoculum was injected into the last proleg using a 26 *G needle* (Terumo, Shibuya City, Japan) and a syringe pump (Thermofisher, Waltham, MA, USA). The inoculum size was checked by plate counting on BHI agar. Saline solution or the ***4f*** compound (based on ¼ MIC or the equivalent of 14 mg/kg for squalamine and 3.5, 14, 1.75, and 7 for ***4f*** in CRAB, CRPA, MRSA, and VRE, respectively) was administered 2 h post-infection. Larvae were incubated at 37 °C and their survival was monitored for 48 h every 2 h, from 16 to 48 h post-infection. Assays were performed in triplicate.

### 3.6. The Preparation of Protein Extracts and Proteomic Analyses

CRAB and CRPA strains were grown in MHBII broth to an OD600 nm of approximately 0.4. On the other hand, MRSA and VRE strains were grown in MH broth to an OD600 nm of about 0.6. Subsequently, the cells were harvested using centrifugation at 8000× *g* for 10 min and resuspended in fresh media containing ¼ MIC of the ***4f*** compound (*n* = 4) or without treatment (control group, *n* = 4). After a 2 h incubation at 37 °C, cells were harvested by centrifugation (8000× *g*, 15 min, 4 °C) and washed once in the extraction buffer (50 mM Tris-HCl pH 7.4, 50 mM Na_2_SO_4_, 15% glycerol). Cells were then resuspended in the same buffer and lysed by using the FastPrep device (MP Biomedicals, Illkirch Graffenstaden, France). Then, the samples were centrifuged at 4 °C and 12,000× *g* for 20 min to collect the supernatant. The protein concentration was measured using the Pierce BCA Protein Assay Kit (Thermoscientific, Rockford, IL, USA). The proteomic analysis of four strains under ***4f*** treatment was then conducted in two distinct proteomic laboratories.

Concerning the CRAB and CRPA samples, the enzymatic digestion of protein extracts and quantitative analysis by mass spectrometry analyses were performed as previously described by Sauvage et al. [65] and Robin et al. [20]. Briefly, 25 μg of proteins were loaded onto a 7% polyacrylamide gel (Acrylamide/Bis-Acrylamide 30% [29:1], Sigma-Aldrich, Steinheim, Germany). Migration was performed for a short period of 90 min at 10–20 mA/gel. After Coomassie blue staining, the revealed protein bands were excised and first immersed in a reductive buffer (5 mM DTT, Sigma-Aldrich) and then an alkylated buffer (20 mM iodoacetamide, Sigma-Aldrich). After washing, the gel bands were digested with 1 μg of trypsin (Promega, Madison, WI, USA) overnight at 37 °C. Then, several steps of peptide extraction were performed using acetonitrile (Fisher, Hampton, NH, USA), and the peptides were dried and stored at −20 °C. The CRAB peptide samples were then analyzed using an LTQ Orbitrap Elite (Thermo Fisher Scientific, Bremen, Germany) coupled to an Easy nLC II system (Thermo Fisher Scientific, Bremen, Germany). Peptide samples from CRPA were analyzed on an Orbitrap Eclipse™ Tribrid™ (Thermo Fisher Scientific, San Jose, CA, USA) coupled to an UltiMate™ 3000 RSLCnano (Thermo Fisher Scientific, San Jose, CA, USA). The detailed chromatography and mass spectrometry analysis protocols are provided in the Supplementary Information part. Raw data after MS analysis were processed using Progenesis LC-MS software (Nonlinear Dynamics, version 4.1.6675.48614, Newcastle, UK). The retention times of all samples were aligned on one reference sample within the experiment. One-way analysis of variance (ANOVA) calculations were performed on aligned and normalized data. The MS/MS spectra from retained peptides were identified using Mascot (Matrix Science, Columbus, OH, USA) against the databases restricted to *A. baumannii* ATCC17978 (http://www.genoscope.cns.fr/, accessed on 12 September 2022) and *P. aeruginosa* PAO1 (https://www.pseudomonas.com/, accessed on 22 April 2021).

For MRSA and VRE samples, 5 µg of each protein extract was prepared using a modified gel-aided sample preparation protocol [66]. Samples were digested with trypsin/Lys-C overnight at 37 °C. For nano-LC fragmentation, protein or peptide samples were first desalted and concentrated onto a µC18 Omix (Agilent, Santa Clara, CA, USA) before analysis. The analysis of MRSA and VRE peptide samples was carried out on a TIMS-TOF Pro mass spectrometer (Bruker Daltonics, Billerica, MA, USA) with a modified nano-electrospray ion source (CaptiveSpray, Bruker Daltonics) coupled with a NanoElute (Bruker Daltonics) ultra-high-pressure nanoflow chromatography system. The detailed chromatography and mass spectrometry analysis protocols are provided in the Supplemental Information. Before post-processing, the samples were analyzed using Preview software (version 3.7.4, ProteinMetrics, Cupertino, CA, USA) to estimate the quality of the tryptic digestion and predict the post-translational modifications present. The result was used for the “bank research/identification” part. The fragmentation pattern was used to determine the sequence of the peptides. Database searching was performed using Peaks XPro software. Updated UniProt *E. faecium* and *S. aureus* databases were used.

To quantify the relative levels of protein abundance between different groups, samples were analyzed using the label-free quantification feature of Progenesis LC-MS (for CRAB and CRPA) and PEAKS XPro software (for MRSA and VRE). A 1.4-fold increase in relative abundance and a *p*-value < 0.05 using a Student’s *t*-test from PERSEUS were used to determine the enriched proteins. Volcano plots were performed with Genoppi [67]. Enrichments in molecular processes, cellular processes, and pathways (KEGG/COG) were performed using the ClueGo app (Version 2.5.9) from Cytoscape software (Version 3.8.2). Enrichments were performed using a Bonferroni step-down method. The mass spectrometry proteomics data were deposited in the ProteomeXchange Consortium via the PRIDE partner repository with the dataset identifier PXD047333 (for CRAB et CRPA) and the iProX partner repository (for VRE and MRSA) with the dataset identifier IPX0007659001.

## 4. Conclusions

In the current context, where the emergence of antibiotic resistance is extremely worrying, the discovery and development of molecules capable of reducing bacterial virulence appears to be an attractive and alternative strategy against MDR pathogens [68,69]. The 6-polyaminosterol derivatives we tested in this study were previously reported for their interesting activities as bactericidal molecules against major resistant pathogens. They have also presented synergistic or additive activities when associated with imipenem, vancomycin, or oxacillin [17]. Here, we highlighted their capacities to prevent and eradicate the biofilms of these pathogens, with two of them (***4e*** and ***4f***) being as efficient as classical antibiotics. The determination of ***4f*** anti-virulence properties in the invertebrate infection model of *G. mellonella* is very promising but requires additional evidence regarding its effectiveness on rodent models or human organoids. Future assessments of ***4f*** anti-virulence activities on a range of clinical strains, including both drug-resistant and susceptible isolates, would also be necessary to confirm its efficacy in the current clinical situation. However, thanks to their potential antivirulence properties, these 6-polyaminosterols could be used as adjuvants in therapeutics to reduce antibiotic dosages and treatment durations, which may contribute to antibiotic resistance mitigation.

## Figures and Tables

**Table 1:** Antibacterial and antibiofilm activities of 6-polyaminosteroid derivatives (mg/L).

		*A. baumannii* CRAB			*P. aeruginosa* CRPA			*E. faecium* VRE			*S. aureus* MRSA		
	IC_50_	MIC	BPC	MBEC	MIC	BPC	MBEC	MIC	BPC	MBEC	MIC	BPC	MBEC
***4a***	52	>64	>128	ND	>64	>128	ND	8	16	>256	32	16	>256
***4b***	25	>64	>128	ND	>64	>128	ND	16	16	>256	16	8	128
***4c***	28	64	128	ND	64	128	ND	8	>32	ND	32	32	ND
***4d***	33	64	128	ND	>64	>128	ND	4	16	>256	16	8	128
***4e***	72	16	64	32	64	128	>256	8	16	64	16	16	32
***4f***	38	32	32	256	16	32	256	16	32	64	16	16	64
***4g***	27	32	128	ND	>64	128	ND	8	16	>256	16	32	ND
***4h***	45	>64	>128	ND	>64	>128	ND	8	16	>256	16	16	>256
***4i***	12	>64	128	ND	>64	>128	ND	8	16	>256	32	32	ND
***4j***	29	>64	128	ND	>64	>128	ND	4	8	>256	16	8	>256
***4k***	17	8	16	>256	64	128	ND	4	8	>256	8	8	>256
***4l***	41	16	32	>256	64	128	ND	16	16	>256	32	16	>256
***4m***	33	64	64	ND	>64	>128	ND	8	16	>256	16	16	>256
***4n***	46	>64	>128	ND	>64	>128	ND	16	16	>256	32	32	>256
***4o***	28	64	128	ND	>64	>128	ND	2	32	>256	8	16	>256
***4p***	15	64	128	ND	>64	>128	ND	8	16	>256	16	16	ND
***4q***	89	16	32	>256	64	64	>256	8	16	>256	8	16	>256
***4r***	38	32	64	128	64	128	ND	8	32	>256	8	32	>256
***SQ***	66	4	8	32	16	32	>256	8	32	>256	2	32	>256

MBECs were determined for derivatives with MIC < IC_50_. “ND” for “not determined”, “SQ” for “squalamine”. MICs and IC_50_ on A549 human lung cells were determined in [17].

**Table 2:** Anti-pyocyanin activity of 6-polyaminosteroid derivatives (mg/L).

	Inhibition > 70%	Inhibition > 40%
***4a***	32	4
***4f***	-	4
***4g***	32	4
***4i***	32	16
***4j***	16	4
***4k***	32	16
***4l***	32	8
***4m***	32	8
***4o***	32	16
***4r***	32	16
***4s***	32	16
**SQ**	-	8

Concentrations leading to an inhibition of over 40 or 70% were determined for derivatives with MIC < IC_50_. “-”: the tested concentration did not lead to an inhibition of over 70%.

**Table 3:** Strains used in this study.

Species	Common Name (Strain Name)	Characteristics	Reference
*S. aureus*	MRSA (MW2)	MET ^R^	[56]
*E. faecium*	VRE (Aus0004)	VAN ^R^	[57]
*A. baumannii*	CRAB (30850)	CARB ^R^ (64 mg/L)	[58]
*P. aeruginosa*	CRPA	CARB ^R^ (64 mg/L)	[17]

MET, methicillin; VAN, vancomycin; ^R^, resistant; CARB, carbapenem.

## Data Availability

The mass spectrometry proteomics data have been deposited to the ProteomeXchange Consortium via the PRIDE partner repository with the dataset identifier PXD047333 (for CRAB et CRPA) and the iProX partner repository (for VRE and MRSA) with the dataset identifier IPX0007659001.

## Supplementary Materials

The following supporting information can be downloaded at https://www.mdpi.com/article/10.3390/antibiotics13010008/s1, Table S1. Proteins differentially expressed in CRAB after ***4f*** treatment. Table S2. Proteins differentially expressed in CRPA after ***4f*** treatment. Table S3. Proteins differentially expressed in VRE after ***4f*** treatment. Table S4. Proteins differentially expressed in MRSA after ***4f*** treatment. Supplementary Information. Chromatography and mass spectrometry analysis methods. Figure S1. Volcano plots of differentially expressed proteins in CRAB, CRPA, VRE, and MRSA groups after 2 h of ***4f*** treatment.
