# Supplementary material for: Antibiofilm and Antivirulence Properties of 6-Polyaminosteroid Derivatives against Antibiotic-Resistant Bacteria
Source: Antibiotics (Basel). 2023 Dec 20;13(1):8. doi: 10.3390/antibiotics13010008 (PMC10812528; doi:10.3390/antibiotics13010008)
Supplement: Supplementary file 1 [file antibiotics-13-00008-s001.zip › Supplementary Material 2.pdf]

Supplementary Material 2

Antibiofilm and antivirulence properties of 6-polyaminosteroid derivatives against antibiotic-resistant bacteria

Delphine Vergoz[1], Hung Le[1], Benoit Bernay[2], Annick Schaumann[1], Magalie Barreau[3], Flore Nilly[3], Florie Desriac[3], Ali Tahrioui[3], Jean-Christophe Giard[4], Olivier Lesouhaitier[3], Sylvie Chevalier[3], Jean Michel Brunel[5], Cécile Muller[3#], and Emmanuelle Dé[1#]

[1] Univ Rouen Normandie, INSA Rouen Normandie, CNRS, Normandie Univ, PBS UMR 6270, F-76000 Rouen, France

[2] Univ Caen Normandie, Proteogen Platform, US EMERODE, F-14000, Caen

[3] Univ Rouen Normandie, Univ Caen Normandie, Normandie Univ, Communication Bactérienne et Stratégies Anti-infectieuses, CBSA UR4312, F-76000 Rouen

[4] Univ Caen Normandie, INSERM, Normandie Univ, DYNAMICURE UMR 1311 F, 14000 Caen, France.

[5] Aix Marseille Univ, INSERM, SSA, MCT, Marseille, France.

[#] **Corresponding authors**: Dr. Cécile Muller, cecile.muller@univ-caen.fr & Pr. Emmanuelle Dé, emmanuelle.de@univ-rouen.fr

Supplemental Information. Chromatography and Mass Spectrometry analysis conditions

CRAB and CRPA: The CRAB peptide samples were then analyzed using an LTQ Orbitrap Elite (Thermo Scientific) coupled to an Easy nLC II system (Thermo Scientific). Peptides were injected into an enrichment column (C18 PepMap100, Thermo Scientific). The separation was performed with an analytical column needle (NTCC-360/100-5-153, NikkyoTechnos, Japan). The mobile phase consisted of H_2O/0.1% formic acid (FA) (buffer A) and ACN/FA 0.1% (buffer B). Tryptic peptides were eluted at a 300 nL/min flow rate using a three-step linear gradient: from 2 to 40% B over 76 min, 40 to 100% B in 4 min, and 10 min at 100% B. The first scan (MS spectra) was recorded in the Orbitrap analyzer (R = 60,000) with a mass range of m/z 400-1800. Then, the 20 most intense ions were selected for MS2 experiments. Singly charged species were excluded from the MS2 experiments. Dynamic exclusion of already fragmented precursor ions was applied for

30 s, with a repeat count of 2, a repeat duration of 30 s, and an exclusion mass width of ± 5 ppm. The precursor isolation width was 2 m/z. Fragmentation occurred in the linear ion trap analyzer with a normalized collision energy 35.

Peptide samples from CRPA were analyzed on an Orbitrap Eclipse™ Tribrid™ (Thermo Scientific) coupled to an UltiMate™ 3000 RSLCnano (Thermo Scientific). Peptides were injected into an enrichment column (C18 PepMap100, Thermo Scientific). The separation was performed with an analytical column (Acclaim PepMapC18 (75 µm x 50 cm, 2µm, 100Å, Thermo Scientific). The buffers for the mobile phase were composed of 100% H_2O/0.1% FA (A) and 80% ACN/20% H_2O/0.1% FA (B). The elution gradient duration was 150 min: from 2.5 to 3.5% B over 3 min, from 3.5 to 25% B in 103 min, from 25% to 40% B in 20 min, from 40% to 100% B in 2 min, 5 min at 100% B, from 100 to 2% B in 1 min and 19 min at 2% B. The flow rate was 300 nL/min. The temperature of the column was set at 40 °C. The maximum injection time was set to 30 ms. The capillary voltage was 1.9 kV. The temperature of the capillary was 275 °C. The analysis was performed in HCD mode. The m/z detection range is 375–1500. The resolution was 120,000 in MS and 15,000 in MS/MS. A top-speed approach was used with a cycle time of 3 sec. Singly charged species were excluded from the MS2 experiments. Dynamic exclusion of already fragmented precursor ions was applied for 40 s, and an exclusion mass width of ± 10 ppm. The ions were selected in the quadruple with an isolation window of 1.6 m/z. For MS2, the maximum injection time was set at 22 msec. Fragmentation occurred with a normalized collision energy of 30.

Raw data after MS analysis were processed using the Progenesis LC-MS software (Nonlinear Dynamics). The retention times of all samples were aligned on one reference sample within the experiment. One-way analysis of variance (ANOVA) calculations was performed on aligned and normalized data. The MS/MS spectra from retained peptides were identified using Mascot (Matrix Science) against the database restricted to *A. baumannii* ATCC17978 (http://www.genoscope.cns.fr/) and *P. aeruginosa* PAO1 (https://www.pseudomonas.com/). Database searches were performed with the following parameters: 1 missed trypsin cleavage site allowed; variable modifications: carbamidomethylation of cysteine and oxidation of methionine. Mass tolerances for precursor and fragment ions were configured at 5 ppm and 0.02 Da for the

Orbitrap Eclipse. In the case of the LTQ Orbitrap Elite, mass tolerances for precursor and fragment ions were set at 10 ppm and 0.35 Da, respectively.

Peptide validation was performed using the Percolator algorithm, and only "high confidence" peptides were retained, corresponding to a 1% false discovery rate. Then, Mascot search results were imported into Progenesis. A minimum of 2 unique peptides were required to consider protein identification and quantification. For each condition, the total cumulative abundance of the protein was calculated by summing the abundances of peptides. Proteins identified with < 2 peptides were discarded.

MRSA and VRE: The chromatography step was performed on a NanoElute (Bruker Daltonics) ultra-high-pressure nanoflow chromatography system. Approximatively 200 ng of each peptide sample was concentrated onto a C_{18} pepmap 100 precolumn (Thermo Scientific) and separated at 50°C onto a reversed-phase Reprosil column packed with 1.6 µm C18 coated porous silica beads (Ionopticks). Mobile phases consisted of 0.1% formic acid, 99.9% water (v/v) (A), and 0.1% formic acid in 99.9% ACN (v/v) (B). The nanoflow rate was set at 300 nl/min, and the gradient profile was as follows: from 2 to 15% B within 15 min, followed by an increase to 25% B within 15 min, and further to 37% within 5 min, followed by a washing step at 95% B and re-equilibration.

MS experiments were conducted on a TIMS-TOF pro mass spectrometer (Bruker Daltonics) with a modified nano-electrospray ion source (CaptiveSpray, Bruker Daltonics). A 1600 spray voltage with a capillary temperature of 180 °C was typically employed for ionizing. MS spectra were acquired in the positive mode in the mass range from m/z 100 to 1700. In the experiments described here, the mass spectrometer was operated in PASEF mode with the exclusion of single-charged peptides. A number of 10 PASEF MS/MS scans were performed during 1.25 seconds from charge range 2-5.

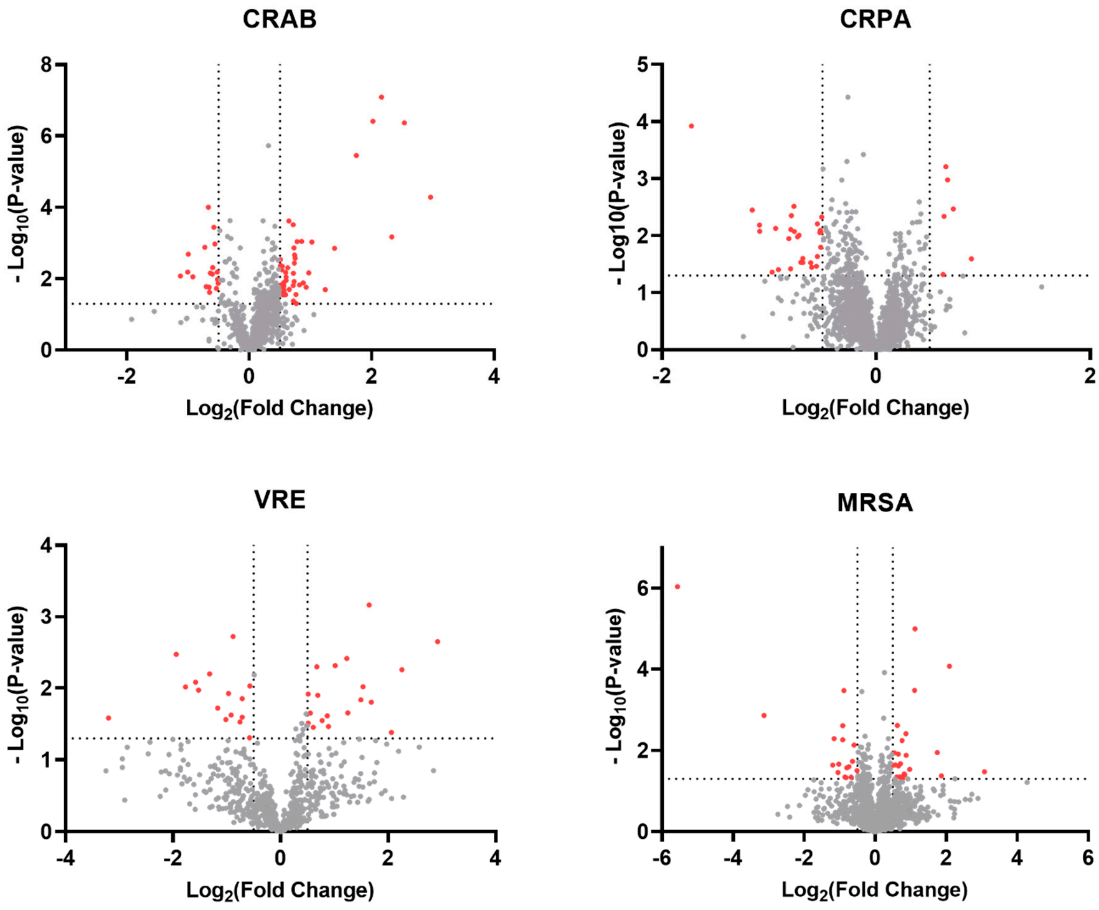

Figure S1. Volcano plots of differentially expressed proteins in CRPA, CRPA, VRE, and MRSA groups after 2h of ***4f*** treatment at a concentration equivalent to ¼ MIC. The horizontal line denotes a p-value < 0.5, while vertical lines signify a Fold-change > 1.4. The red dots represent proteins with significant differential expression, while the gray dots indicate proteins where differences in abundance did not reach statistical significance.
